# The Effect of Cysteine on the Removal of Cadmium in Paddy Soil by Combination with Bioremediation and the Response of the Soil Microbial Community

**DOI:** 10.3390/toxics13010022

**Published:** 2024-12-29

**Authors:** Emmanuel Konadu Sarkodie, Kewei Li, Ziwen Guo, Jiejie Yang, Yan Deng, Jiaxin Shi, Yulong Peng, Yuli Jiang, Huidan Jiang, Hongwei Liu, Yili Liang, Huaqun Yin, Xueduan Liu, Luhua Jiang

**Affiliations:** 1 School of Minerals Processing and Bioengineering, Central South University, Changsha 410083, China; emmasarkk@gmail.com (E.K.S.); 15116475510@163.com (K.L.); kidgzw@hotmail.com (Z.G.); jiejieyang@csu.edu.cn (J.Y.); 18874761977@163.com (J.S.); 225611009@csu.edu.cn (Y.P.); 235611001@csu.edu.cn (Y.J.); hongweiliu@csu.edu.cn (H.L.); liangyili6@csu.edu.cn (Y.L.); yinhuaqun_cs@sina.com (H.Y.); xueduanliu@csu.edu.cn (X.L.); 2 Key Laboratory of Biometallurgy of Ministry of Education, Central South University, Changsha 410083, China; 3 Hunan Agricultural Biotechnology Research Institute, Hunan Academy of Agricultural Sciences, Changsha 410125, China; dengyan202103@163.com (Y.D.); jianghuidan@hunaas.cn (H.J.)

**Keywords:** paddy soil remediation, Cd pollution, bioremediation, cysteine, microbial community

## Abstract

Bioremediation is widely recognized as a promising and efficient approach for the elimination of Cd from contaminated paddy soils. However, the Cd removal efficacy achieved through this method remains unsatisfactory and is accompanied by a marginally higher cost. Cysteine has the potential to improve the bioleaching efficiency of Cd from soils and decrease the use cost since it is green, acidic and has a high Cd affinity. In this study, different combination modes of cysteine and microbial inoculant were designed to analyze their effects on Cd removal and the soil microbial community through the sequence extraction of Cd fraction and high-throughput sequencing. The results demonstrate that the mixture of cysteine and the microbial inoculant was the best mode for increasing the Cd removal efficiency. And a ratio of cysteine to microbial inoculant of 5 mg:2 mL in a 300 mL volume was the most economically efficient matching. The Cd removal rate increased by 7.7–15.1% in comparison with the microbial inoculant treatment. This could be ascribed to the enhanced removal rate of the exchangeable and carbonate-bound Cd, which achieved 94.6% and 96.1%, respectively. After the treatment, the contents of ammonium nitrogen (NH_3_–N), total phosphorus (TP), available potassium (AK), and available phosphorus (AP) in the paddy soils were increased. The treatment of combinations of cysteine and microbial inoculant had an impact on the soil microbial diversity. The relative abundances of *Alicyclobacillus*, *Metallibacterium*, and *Bacillus* were increased in the paddy soils. The microbial metabolic functions, such as replication and repair and amino acid metabolism, were also increased after treatment, which benefitted the microbial survival and adaptation to the environment. The removal of Cd was attributed to the solubilizing, complexing, and ion-exchanging effects of the cysteine, the intra- and extracellular adsorption, and the production of organic acids of functional microorganisms. Moreover, cysteine, as a carbon, nitrogen, and sulfur source, promoted the growth and metabolism of microorganisms to achieve the effect of the synergistic promotion of microbial Cd removal. Therefore, this study underscored the potential of cysteine to enhance the bioremediation performance in Cd-contaminated paddy soils, offering valuable theoretical and technical insights for this field.

## 1. Introduction

Cadmium (Cd) pollution is considered a serious environmental concern due to its undesirable impacts. Cd is a highly phytotoxic, non-redox-active, and bioaccumulative heavy metal that not only destroys the ecological environment but also poses a significant threat to human health. Rice (*Oryza sativa* L.) serves as a staple food for over half of the global population, particularly in Asia. It has genes related to Cd absorption and transport, such as OsNRAMP5, which leads to a high Cd uptake amount in rice and high accumulation in its grain [1]. Thus, Cd enters the food chain, endangering the health of humans and other organisms [2,3]. Paddy soils have been contaminated with Cd [4,5,6], such as in Japan, India, and China, as a result of anthropogenic activities, such as mining, smelting, industrial waste, low-quality phosphate fertilizers, pesticide application, and atmospheric pollutant deposition [7,8]. Therefore, there is an urgent need to remediate Cd-contaminated paddy soils and reduce the Cd uptake by rice.

Recently, a variety of techniques have been utilized to remediate Cd-contaminated paddy soils, including lime application, adding passivation material, soil washing, hyperaccumulator extraction, low-Cd-accumulation rice variety planting, and horticultural regulation [9,10,11,12,13]. Among these methods, bioleaching was proved to be a promising remediation technology for heavy-metal-polluted soils due to its characteristic of low cost, ease of operation, permanent removal, and eco-friendliness [14]. R. Kumar and R. Nagendran used heterotrophic and acidophilic Acidithiobacillus thiooxidans for bioleaching heavy metals from contaminated soils; these microbes were able to oxidize inorganic sulfur compounds and ferrous ions to produce sulfuric acid and ferric ions, which are the main compounds leading the leaching processes. The results showed that the bioleaching process could transform the heavy metals to not easily mobile fractions in soil and acted as an efficient reagent for removing heavy metals from the soil [15]. In addition, it has been reported that *Deinococcus radiodurans* can remove Cd and Pb from paddy soil to prevent the translocation of and damage from Cd or Pb in rice [16]. The extremophile red microalgal strain *Galdieria sulphuraria* was also found to remove Cd, Pb, Ni, and Zn from acidic aqueous solutions [17,18]. Deng et al. studied bioleaching heavy metals from contaminated soil by *Penicillium chrysogenum*. This heterotrophic microorganism could produce metabolites, including organic acids and other substances containing functional groups (–NH_2_, –OH, –CHO, –CO); the maximum bioleaching efficiencies for Pb, Zn, Cd, and Cu were 100%, 70.76%, 35.80%, and 9.38%, respectively [19]. In our previous research work, a mixed consortium of autotrophic and heterotrophic bacteria (microbial inoculant) was artificially constructed to diminish the toxicity of low-molecular-weight organic matter on acidophilic bacteria and increase their environmental adaptability [14]; the results indicate that the Cd removal efficiency in paddy soils could increase from 0.74% to 32.09%. Although bioleaching had a good performance for Cd removal from paddy soils through our developed microbial inoculant, the removal of Cd from paddy soils was still unsatisfactory due to Cd re-adsorption onto soil particles through ion exchange and organic matter complexation [20]. Furthermore, a lot of water, electricity, and nutrients are consumed during the microorganism culture, resulting in a high cost of Cd remediation in paddy soils.

Cysteine, as a non-toxic and low-cost amino acid, has three types of functional groups, namely, –SH, –NH_2_, and –COOH, making it a good complexing agent for heavy metals, where -SH can accelerate the release of Cd in the soil through complexation [21,22], making it a good complexing agent for heavy metals. In particular, –SH has non-bonded lone pairs that preferentially coordinate with Cd^2+^ according to the hard–soft acid–base (HSAB) principle [23]. Therefore, cysteine can be effectively used to leach Cd from paddy soils, preventing Cd re-adsorbing onto soil particles. Recently, the selective removal ability of cysteine to Cd in water has been reported [24]. For instance, Fan et al. synthesized an Fe_3_O_4_/MOF/cysteine composite to remove Cd from wastewater; the results show that the Cd removal efficiency of Fe_3_O_4_/MOF/cysteine (98%) was much higher than Fe_3_O_4_/MOF (62%). The reason for the higher efficiency of Fe_3_O_4_/MOF/cysteine was attributed to the coordination between the –SH in cysteine and Cd^2+^ [25]. Furthermore, the low pH of the solution and the reducibility of the cysteine can facilitate the dissolution of Cd from the carbonate-binding, iron/manganese-binding, and sulfide-binding fractions and the desorption from soil organic matter [26]. This indicates that cysteine could play a good role in Cd removal in paddy soils. In addition, cysteine, as a microbial growth factor, can promote microbial metabolic activity. For example, Göbbels et al. revealed by transcriptome analyses that *M. thermoacetica* can utilize cysteine as a source of carbon and energy to drive metabolism under Cd and blue light stress, and then protect the cells from potential toxic effects through mechanisms such as enzyme inhibition or oxidative stress [27].

Consequently, we hypothesized that the addition of cysteine to the microbial inoculant could improve the bioleaching efficiency of Cd from polluted paddy soils and decrease the amount and use cost of the microbial inoculant. Then, based on determining the optimal combination mode of cysteine and the microbial inoculant, this study clarified the transformation behavior of Cd in paddy soils under the mixture of cysteine and the microbial inoculant and revealed the renewal pattern of the microbial community structure in paddy soils after the mixture leaching.

## 2. Materials and Methods

### 2.1. Soil Sample Collection

In this study, paddy soils contaminated with Cd were collected from the “Chang–Zhu–Tan” area in the Hunan Province, and three sampling sites were selected. Samples were obtained from (i) an experimental field in Beishan town, Changsha County, Hunan Province (28°26′11″ N, 113°03′14″ E), and designated as S1; (ii) an experimental field in Zhongjiawan in Qingshan Village, Liling City, Hunan Province (27°31′25.29″ N, 113°14′21.03″ E), and designated as S2; and (iii) an experimental field in Lijiaba in Qingshan Village, Liling City, Hunan Province (27°43′25.98″ N, 113°35′16.71″ E), and designated as S3. Following the collection, the soil samples were placed in free Ziplock bags, and impurities, such as rocks and plant residues, were removed before being transported to the laboratory. The soil samples were air dried, passed through a 10-mesh nylon sieve, and then stored at 4 °C and –80 °C for the subsequent analyses of the physicochemical properties and microbial communities, respectively.

### 2.2. Soil Physicochemical Properties Analysis

The soil texture was determined by using the hydrometer method in the lab according to the LY/T 1225–1999 standards [28] and according to the proportions of sand (2–0.05 mm), silt (0.05–0.002 mm), and clay (<0.002 mm) [29]. The soil pH was measured by a digital pH meter (PHS–3E, Leici, China) in a deionized water extract (soil-to-water 1:2.5, *w*/*v*) according to the NY/T 1377–2007 standards [30]. The soil organic matter (SOM) content was measured by the potassium dichromate (K_2_Cr_2_O_7_) digestion method based on NY/T 1121.6–2006 [31]. The ammonium nitrogen (NH_3_–N) and nitrate nitrogen (NO_3_–N) contents were determined by using the indophenol blue method and the calcium chloride method (HJ 634–2012) [32]. The total nitrogen (TN) was determined by alkaline potassium persulfate digestion and ultraviolet spectrophotometry according to HJ 636–2012 [33]. The available phosphorus (AP) was determined using the sodium hydrogen carbonate solution–Mo–Sb anti-spectrophotometric method (HJ 704–2014) [34]. The available potassium (AK) was measured by the ammonium acetate solution extraction method and flame photometer method (NY/T 889–2004) [35]. The total phosphorus (TP) was extracted based on HJ 632–2011 by the alkali fusion–Mo–Sb anti-spectrophotometric method [36]. The total content of Cd in the soil was determined by digesting 0.500 g fine soil samples in mixed acids (HNO_3_–HF–HClO_4_) according to GB/T 17141–1997 [37]. And then, the concentration of Cd in the digested solution was measured by inductively coupled plasma optical emission spectrometry (ICP–OES, Agilent 5100 SVDV, Agilent Technologies, Inc., Santa Clara, CA, USA) [38]. The Cd fractions in different soils before and after leaching, including the exchangeable fraction (F1), carbonates bound fraction (F2), iron and manganese oxides bound fraction (F3), organic matter bound fraction (F4), and residual fraction (F5), were conducted using the modified Tessier sequential extraction procedure (Table S1) [39].

### 2.3. Leaching Experiment

In this study, cysteine (98% purity) was purchased from Huaxiang Kejie Biotechnology Co., Ltd, Wuhan, China, and the microbial inoculant was obtained from our previous research [14]. Various combination modes of cysteine and microbial inoculant were adopted to research the Cd leaching performance from the paddy soils. The leaching experiment was conducted three times for 45 min each. The experimental group design is shown in Table 1.

In this experiment, a 300 mL cysteine (0.3 g–4.5 g) solution or microbial inoculant was added into beakers T1–T8, ensuring the initial pH of the reaction system was maintained at about 2. In T9, the mixture contained 0.1 g cysteine and 100 mL microbial inoculant. Before the leaching, the solution was diluted 3-fold with water to 300 mL. After obtaining the best combination mode of cysteine and microbial inoculant, the effects of different ratios of the microbial inoculant and cysteine in the mixture on Cd leaching from paddy soils were researched. The experimental group design is shown in Table 2.

For each of the leaching experiments, 100 g of soil was added in a 1 L beaker, followed by the addition of an appropriate volume of solution with a solid–liquid ratio of 1:3 (*w*/*v*). Subsequently, the 1 L beaker was placed on a six-unit electric agitator platform (JJ–3A, Jinyi Instrument Technology Co., Ltd., Jiangsu, China) and shaken at 400 rpm for 45 min. After this, the suspended samples were taken and centrifuged at 8000 r/min for 5 min, and the supernatants were filtered through a 0.22 mm filter. The content of Cd in the filtrate was analyzed by using ICP–OES (Agilent 5100 SVDV, America Agilent Technologies). Afterward, 10 g of the washed soils was taken and stored at –80 °C for the microbial community analysis. For the soil physicochemical properties analysis, the leftover residue was then air-dried at room temperature to a consistent weight and then crushed to pass through a 100-mesh (0.15 mm) nylon sieve. All experiments were conducted in triplicate. The Cd removal rates in these paddy soils were calculated using Equation (1):

*R* = (TCd_o_ − TCd_t_)/TCd_o_ × 100% (1)

where *R* (%) represents the Cd removal rate, TCd_o_ (mg/kg) represents the total Cd content in the paddy soil before treatment, and TCd_t_ (mg/kg) represents the total Cd content in the paddy soil after treatment.

### 2.4. DNA Extraction and High-Throughput Amplicon Sequencing

DNA kits (D4015, Omega Bio–tek Inc., Norcross, GA, USA) were used to extract the genomic DNA from the pristine paddy soil and leached paddy soil in S2. The bacterial oligonucleotide primer pairs 515 F (5′–GTGYCAGCMGCCGCGGTAA–3′) and 806 R (5′–GGACTACNVGGGTWTCTAAT–3′) were used to amplify the V4 region of the 16S rRNA gene via a polymerase chain reaction (PCR). The PCR amplification was performed using 25 μL template DNA, 12.5 μL PCR premixes, and 2.5 μL primers, and the reaction liquid was 25 μL. The PCR protocol was an initial denaturation step at 98 °C for 30 s, followed by 32 cycles comprising denaturation at 98 °C for 10 s, annealing at 54 °C for 30 s, and extension at 72 °C for 45 s. A final extension was performed at 72 °C for a duration of 10 min. The PCR results were validated using 2% agarose gel electrophoresis. High-throughput sequencing was subsequently conducted on the Illumina NovaSeq PE250 platform by Lianchuan Biotechnology Co., Ltd. (Hangzhou, Zhejiang, China), and the obtained raw data were aptly analyzed. The trimmed FASTQ data were converted to the FASTA format, and UPARSE was used to cluster sequences with a 97% similarity. The operational taxonomic units (OTUs) were obtained and annotated by the Ribosomal Database Project (RDP) classifier.

### 2.5. Data Processing and Statistical Analysis

Illumina NovaSeq was used to sequence the samples in accordance with the instructions. The samples were assigned paired-end reads based on their unique barcodes and truncated by cutting the barcode and primer sequence. In order to obtain clean tags of high quality, the raw data were quality-filtered using fqtrim (v0.94) under specific filtering conditions, and using Vsearch software (version 2.3.4), the chimeric sequences were filtered. Feature tables and feature sequences were obtained by denoising the reads into amplicon sequence variants (OTUs) using DADA2 and clustering by dereplication. Based on the SILVA (release 132) classifier, feature abundances were subsequently normalized using the relative abundances. The alpha diversity indices, including Chao 1, observed species, Goods coverage, Shannon, and Simpson, were analyzed using the QIIME2 software package. QIIME2 was used to calculate the beta diversity and Venn diagrams. The data obtained from triplicate experiments are given as mean ± standard deviation values. Statistical analysis was conducted with Minitab 17.0 software (Minitab Inc., Philadelphia, Pennsylvania, USA) to identify the differences in the soil properties. The Cd removal efficiencies between the treatments were analyzed using one-way analysis of variance (ANOVA). Fisher’s least significant difference test (LSD) was used to compare the significance (*p* < 0.05) between the means. Other graphs were generated by Origin 2021.

## 3. Results and Discussion

### 3.1. Soil Physicochemical Properties of Original Paddy Soil

The total Cd contents were 0.933 mg/kg, 1.035 mg/kg, and 5.00 mg/kg in S1, S2, and S3, respectively. This indicates that the sampled soils were slightly contaminated with Cd. The Cd pollution was caused by rainwater washing from the surrounding mines and fertilization in the fields [37]. The reason for the Cd levels exceeding the allowable limit in the studied soils could have been due to the contaminated water for irrigation, atmospheric sedimentation, and mass applications of potentially toxic pesticides and agricultural fertilizer [40,41]. The fraction proportions of Cd in S1 were 64.3% (F1), 14.7% (F2), 11.7% (F3), 2.5% (F4), and 6.8% (F5). In S2, the proportions of F1, F2, F3, F4, and F5 were 46.4%, 7.4%, 7.1%, 4.1%, and 35.1%, respectively. The order of the proportions of each fraction in S3 was F1 (60.7%) > F2 (10.9%) > F3 (10.5%) > F5 (9.2%) > F4 (8.6%). It could be realized that the Cd in all soil samples had high proportions of F1 and F2, indicating a high potential for Cd mobilization and migration and easiness for plants to absorb it. It is also worth noting that the comparatively higher F5 fractions in S2 imply that the removal of Cd would be inevitably more difficult. According to the texture triangle of the United States Department of Agriculture (USDA) soil classification system, S1 had 66.7% sand, 18.1% clay, and 15.2% silt, and hence, was classified as sandy loam. S2 had 34.6% sand, 42.9% silt, and 23% clay and was also classified as loam. However, S3 had 24.1% sand, 43.9% silt, and 32% clay and was classified as clay loam. Other representative physicochemical properties of the sampled soils are presented in Table 3 and Table 4. As seen, the soil pH was strongly acidic, with the values of 3.92, 3.80, and 4.33 in S1, S2, and S3, respectively. The strong acidity of the soil might be attributed to the characteristics of red soil and N fertilizer application [42]. Soil acidification, which promotes the activation of most heavy metals, has been a key factor in excess Cd in crops [43]. SOM plays a vital role in controlling the mobility of heavy metals in the soil. It may decrease the available concentration of heavy metals in the soil through the adsorption or complexation process [44]. The contents of SOM were 0.52%, 0.85%, and 0.51% in S1, S2, and S3, respectively. This indicated that the mobility of Cd in S2 was lower than those in S1 and S3, potentially resulting in difficulty of Cd removal. The NO_3_–N was the highest in S2 (157.12 mg/kg) and the lowest in S1 (72.42 mg/kg). Furthermore, NH_3_–N was significantly higher in S2 (114.43 mg/kg) and lower in S3 (8.34 mg/kg). The TN was the highest in S3 (2.02 g/kg), followed by S2 (1.60 g/kg) and S1 (1.20 g/kg). The TPs in the soil were 0.40 g/kg, 0.30 g/kg, and 0.40 g/kg in S1, S2, and S3, respectively. The AP in S2 (4.28 mg/kg) was significantly lower as compared with S1 (25.54 mg/kg) and S3 (26.26 mg/kg). Moreover, the AK was also significantly higher in S2 (173.95 g/kg) than in S1 (83.0 g/kg) and S3 (67.2 g/kg).

### 3.2. Effect of Combination Mode of Cysteine and Microbial Inoculant on Cd Removal

**Figure 1:**
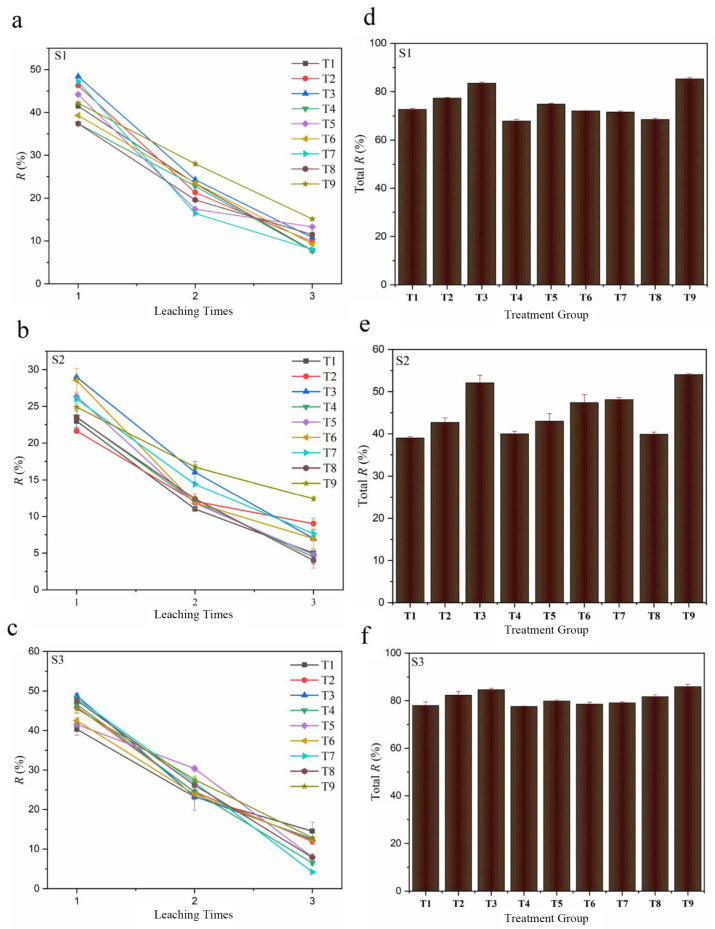
Removal rate of Cd from contaminated paddy soils in the different combination mode of cysteine and microbial inoculant (**a**–**c**); the total removal rate calculated by soil digestion (**d**–**f**).

The Cd removal efficiencies in the polluted paddy soils by the different combination modes of cysteine and the microbial inoculant are shown in Figure 1a–c. As seen, the process mainly involved two distinct stages for the removal of Cd from S1–S3. The removal efficiency was higher during the first time and subsequently declined in the second and third times. This might be ascribed to the Cd in the soil samples having a high proportion of F1 and F2, which resulted in a quick Cd release triggered by the cysteine dissolution in the first round of leaching [45]. Then, the remaining stable fractions of Cd were removed at a slower rate in the subsequent leaching due to their insolubility property [46]. As shown in Figure 1d–f, all combination modes showed significantly less efficiency of Cd removal in S2 than S1 and S3. This might have been due to S2 having the highest proportion of F5 (35.1%) in all the soil samples, which led to more difficult Cd removal. In addition, the Cd removal rates of groups with cysteine alone (T2) were 77.4%, 42.7%, and 83.5% in S1, S2, and S3, respectively. However, the removal rates of groups with the microbial inoculant alone (T1) were 72.7%, 39.0%, and 78% in S1, S2, and S3, respectively. The removal rate of Cd by T2 was superior to that of T1. This was mainly due to the fact that the –SH of cysteine could form a soluble complex with Cd and inhibited the re-adsorption of Cd on the soil particle surface, thus achieving the higher Cd removal ability [22].

As shown in Figure 1d–f, the removal efficiencies in all soil samples could be described in the following order: T9 > T3 > T2 > T5 > T7 > T6 > T1 > T8 > T4. Moreover, when comparing T1–T8, T4 had the lowest Cd removal rate in all the soil samples, while T3 showed the highest removal rate. This indicates that the combination mode of cysteine and the microbial inoculant had a significant effect on the Cd removal from the paddy soils. Hence, Cd leaching could be enhanced by a suitable combination approach. Further analysis found that the mixture of cysteine and the microbial inoculant solution (T9) was more effective at removing the Cd from the contaminated paddy soils. The removal rates of T9 were 85.2%, 54.1%, and 85.9% in S1, S2, and S3, respectively. This indicates that mixing cysteine and the microbial inoculant was the optimal mode of application. In addition to having the highest Cd removal rate, T9 also had the lowest usage amount of cysteine and microbial inoculant. Therefore, cysteine could directly promote the leaching of Cd from the paddy soil by the microbial inoculant. Also, the mixed application approach was evidently a better choice for the Cd removal in the polluted paddy soils. Some studies also demonstrated the promoting effect of cysteine on the bioleaching process, such as Ni, Zn, and Cu [47,48,49]. For instance, a study found that 0.2 g/L cysteine added into a Ni–Cu sulfide bioleaching system could increase the liquid-phase redox potential, cell density, solution acidity, and surface zeta potential, which resulted in an enhanced recovery rate of Ni and Cu [50]. One reason for the promoting effect of cysteine was that cysteine can be used as a sulfur source or substrate for microorganisms to promote their growth and metabolism, and then more organic acids or other metabolites containing active functional groups are produced to desorb Cd from the soil [51]. In addition, the complexation of functional groups, such as –SH, and the reducibility of cysteine can promote Cd leaching from soils by the microbial inoculant [26]. Meanwhile, the lower pH caused by cysteine can provide a better growth condition for acidophilic microorganisms in the microbial inoculant and promote Cd dissolution from paddy soils [52]. Thus, due to the promoting effect of cysteine onto the microbial inoculant, mixing cysteine could decrease the usage amount and use cost of the microbial inoculant and increase its Cd removal rate from the paddy soils.

### 3.3. Effect of Additive Amount of Cysteine in the Mixture on the Cd Removal in the Paddy Soil

**Figure 2:**
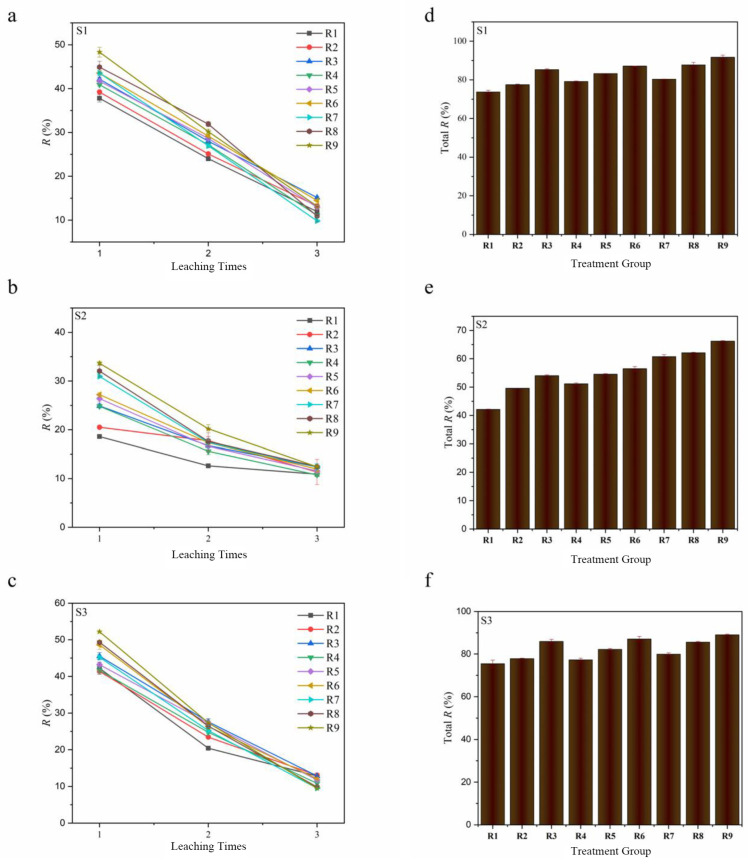
Removal rates of Cd from contaminated paddy soils after leaching with different additive amount of cysteine and microbial inoculant in the mixture (**a**–**c**); the total removal rates calculated by soil digestion (**d**–**f**).

The dosage of cysteine in the mixture plays a crucial role in determining both the removal rate of Cd in the paddy soil and the associated treatment cost. Therefore, additional research was undertaken to investigate the impact of varying the cysteine concentration in the mixture on the removal of Cd. As depicted in Figure 2a–c, the removal rate of Cd from the paddy soils gradually increased with the escalating dosage of the microbial inoculant and cysteine during the initial leaching process. The maximum dosage of R9 yielded the most effective leaching results in S1, S2, and S3, with 91.7%, 66.25%, and 89.05%, respectively. In contrast, the minimum dosage of R1 displayed the weakest Cd removal efficiencies during the initial leaching rounds, with 73.75%, 42.1%, and 75.45% in S1, S2, and S3, respectively. This might be mainly attributed to the lower pH value brought about by the higher additions, which facilitated the release of Cd from the soil by dissolution. Meanwhile, more cysteine not only provided more Cd complexation sites but also served as a carbon and energy source to promote more active microbial activities, which was conducive to the rapid leaching of Cd [53,54,55]. However, some groups with lower dosages also showed higher removal rates in the two subsequent leachings. For example, the Cd removal rate of R8 was higher than that of R9 in the second leaching in S1, and the Cd removal rate of R1 was also higher than that of R9 in the third leaching in S3. This suggests that although low dosages could not achieve faster Cd removal in the first leaching, sufficient leaching time and continuous leaching reagent supplementation could still facilitate subsequent leaching.

Figure 2d–f illustrates the elimination rate of Cd from paddy soils achieved through soil digestion. The final Cd removal from paddy soils was significantly influenced by the additive dosage of cysteine and microbial inoculant in the mixture. When maintaining a constant level of cysteine amount in the mixture, a higher concentration of the microbial inoculant resulted in improved Cd removal. Similarly, an increase in the amount of cysteine promoted Cd removal at a consistent concentration of the microbial inoculant in the mixture. The maximum removal rates of 91.7%, 66.25%, and 89.05% in S1, S2, and S3, respectively, were achieved through treatment R9, whereas the minimum removal rates of 73.75%, 42.1%, and 75.45% were observed with treatment R1 in S1, S2, and S3, respectively. To further assess the economic benefits of mixtures with different dosages, the cost of the reagent for removing Cd per unit mass from the soil was calculated. As shown in Figure S1, the additive dosage of cysteine and microbial inoculant in the mixture had a significant effect on the economic benefit. Comparing R1–R3, R4–R6, and R7–R9, it can be seen that when the amount of microbial inoculant was the same, increasing the dosage of cysteine could improve the economic benefit. However, when the dosage of cysteine was the same (e.g., R1, R4, R9), a higher additive amount of microbial inoculant made the cost higher. It could be found that R3 was the most economically efficient treatment group. Notably, R3 also attained relatively high Cd removal rates of 85.32%, 54.05%, and 85.95% in S1, S2, and S3, respectively. Thus, taking into comprehensive consideration the removal efficiency and economic cost, R3 is the more suitable selection that can be employed for the removal of Cd from contaminated paddy soils in actual applications.

### 3.4. Changes in the Cd Fraction in Paddy Soils After Leaching with the Mixture of Cysteine and the Microbial Inoculant

**Figure 3:**
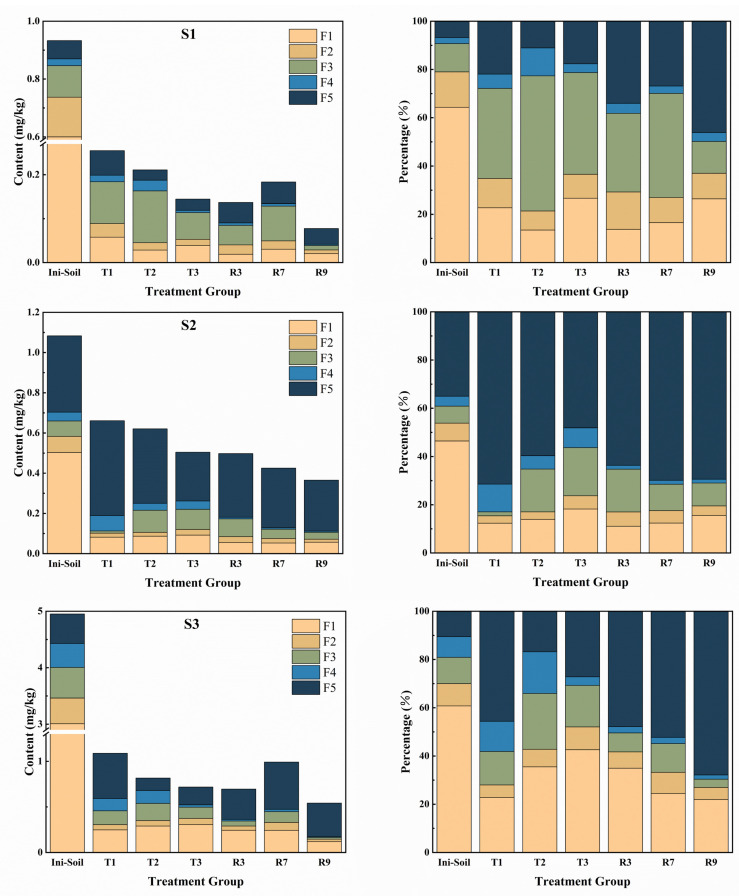
The contents and percentages of different fractions of Cd in paddy soils before and after leaching.

After leaching with the mixture of cysteine and the microbial inoculant effectively reduced the total content of Cd in the paddy soil. However, Cd interacts with soil components and usually exists as multiple bound fractions [56]. In order to gain insight into the transformation of Cd fractions during leaching and the toxicity of residual Cd, the fractions of Cd before and after leaching by T1, T2, T3, R5, R7, and R9 were determined by using the Tessier sequential extraction method. As seen in Figure 3, there were significant reductions in the contents of the F1 and F2 fractions in S1 after all the treatments. Compared with all the treatments, R9 led to the largest reduction in the contents of the F1, F2, F3, and F4 fractions, with 96.60%, 94.00%, 90.65%, and 87.83%, respectively. Meanwhile, T3 removed the most content from F5, with 59.80%. The mixture of cysteine and the microbial inoculant in R3 and R9 proved to be more effective at removing the bioactive state of Cd (F1 + F2) than the other treatments, where it achieved removal rates of 94.56% and 96.11%, respectively. Furthermore, when comparing R7 and R9, it was seen that the removal of all the fractions of Cd was significantly enhanced with increased cysteine dosages. This indicates that the microbial inoculant and cysteine synergistically facilitated the removal of Cd. Furthermore, among all the treatment groups, only F3 in T2 demonstrated an increase after leaching. This was consistent with our previous study, which showed that the reducing ability of cysteine caused Fe–Mn oxides to undergo dissolution followed by recrystallization during leaching [26]. In S2, F1 and F2 showed a decrease in all treatment groups, especially in R9, where the highest removal rates were reached at 88.75% and 81.79%, respectively. Furthermore, the content of F5 witnessed a notable increase of 34.86% in T2, whereas it demonstrated a consistent decrease in all the remaining treatment groups. For S3, all the fractions of Cd showed a decreasing tendency in all treatment groups. Among them, F1, F2, F3, and F4 reached the maximum removal in R9, with 96.04%, 94.05%, 96.58%, and 97.75%, respectively, whereas F5 was the most removed in T2, with 73.66%.

In addition, the sequential treatment (R3, R7, R9) for the total removal of Cd was superior to the individual treatment (T1, T2). However, the superiority of T3 at removing specific fractions of Cd was largely influenced by the soil properties. For instance, in S1, T3 had greater F3 and F4 removal than T1 and T2, while T3 showed more removal of F5 in S2. This might be attributed to the fact that the contact between the microorganisms and the cysteine remaining in the soil during the continuous leaching process facilitated Cd leaching, but this incomplete mixing could not fully utilize the synergistic effect of cysteine and the microbial inoculant. When further comparing the mixed and sequential applications, it was obvious that the mixed application was more advantageous in the removal of various fractions of Cd. In particular, R9 had the highest total Cd reduction in all soils, which was attributed to its efficient removals of F1, F2, F3, and F4. This was mainly attributed to the complexation sites for Cd provided by cysteine that prevented Cd from being adsorbed back onto the soil particles, while cysteine promoted bacterial activity, both of which synergistically contributed to the efficient leaching of Cd [57]. The distribution of Cd fractions in the soil after leaching was likewise one of the important indicators for assessing the effectiveness of remediation. After leaching with all the treatments, the proportion of F1 + F2 decreased significantly. In addition, as the most stable fraction in the environment [58], the proportion of F5 increased in all the treatment groups. Among them, F5 reached the lowest percentages in the soil after the R9 treatment, which were 46.15%, 69.41%, and 67.87% in S1, S2, and S3, respectively. This indicates that not only the total Cd content decreased after leaching but also the environmental risk of residual Cd decreased by leaching with the mixture of cysteine and the microbial inoculant.

### 3.5. Changes in the Physicochemical Properties of Paddy Soils After Leaching by the Mixture of Cysteine and the Microbial Inoculant

Heavy metal removal is greatly influenced by soil physicochemical properties. Furthermore, physicochemical properties also influence the utilization of soil after remediation [59]. The soil physicochemical properties measured before and after the treatment are presented in Table 3 and Table 4. As seen, the physicochemical properties of the Cd-contaminated paddy soil changed after the applications of all the treatments. As a key determinant in controlling the migration of Cd in the soil, the pH value decreased significantly after all the treatments as compared with the original soil [60]. This means that the soil pH must be restored in a timely manner after the leaching treatment by applying lime and organic fertilizers to prevent soil acidification from inhibiting normal plant growth. Among the different groups, the pH value after the treatment with R3 showed the highest levels with 3.28, 3.49, and 4.00 in S1, S2, and S3, respectively. This was mainly attributed to the low dosages of the cysteine and microbial inoculant. The SOM content was decreased by all the treatments, which was consistent with the studies by Zupanc et al. [61]. This might be attributed to the physical disruption of the soil structure during leaching caused by the stirring, which subsequently led to the washing away of some small molecular organic acids or organic colloids [62]. In addition, cysteine and the microbial inoculant might accelerate the decomposition of organic matter by promoting the activity and metabolism of soil microorganisms [62,63].

After undergoing almost all the treatments, the TN content witnessed varying degrees of reduction. However, there was a notable increase in the NH_3_–N content across all the treatments and soil types. The maximum increases in this value were 77, 41, and 51 times in S1, S2, and S3, respectively. The NO_3_–N content showed different change trends in the different soils. For example, all treatments significantly elevated the NO_3_–N content in S1. Meanwhile, in S2 and S3, the changes in the NO_3_–N contents showed different performances in the different treatment groups. Cysteine is a nitrogenous organic substance. When added into soils, it can act as a nitrogen source and energy source for microorganisms, activating microorganisms in the soils to decompose organic matter and then release NH_3_–N [64]. In addition, the TP, AK, and AP showed increasing trends after the treatments. At S1, the most significant increments in the TP were observed for R9, T1, and T3, with respective increases of 74%, 127%, and 26%. With respect to S1, S2, and S3, the increases in the AK were notably observed in the T3, T1, and T3 groups. It could also be observed that the contents of TP, AK, and AP exhibited notable increases in all soil samples following the application of the R3 treatment. This suggests that the combined utilization of cysteine and microbial inoculant treatments could also contribute significantly to the soil fertility after remediation, specifically with regard to soil nutrients like phosphorus and potassium.

### 3.6. Influence of the Mixture Leaching on the Microbial Community Structure of Paddy Soil

#### 3.6.1. Alpha Diversity

**Figure 4:**
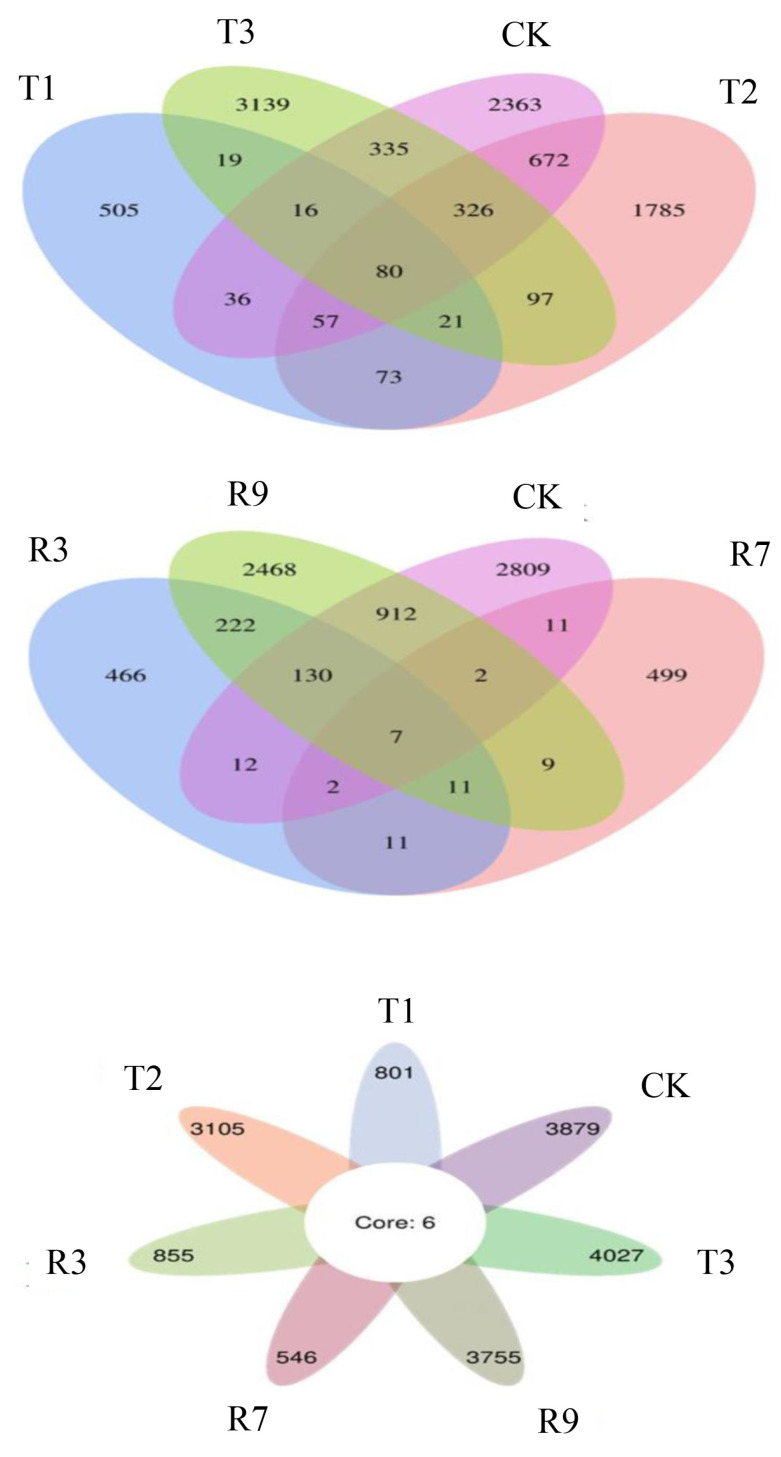
Venn diagram among CK, T1, T2, T3, R3, R7, and R9.

The impacts of leaching on the paddy soil microbial community were analyzed using a high-throughput sequencing technology. As shown in Figure 4, the Venn diagrams visually illustrated the overlaps and distinctions between the original paddy soil (CK) and the paddy soils subjected to various leaching treatments (T1, T2, T3, R3, R7, R9) at the OTU level, with a total of 16,974 OTUs obtained in all the samples. As shown in Figure 4 upper image, 80 OTUs overlapped across four groups. Notably, T3 demonstrated a unique richness with 3139 exclusive OTUs, which surpassed the numbers of unique OTUs identified in T1 (505), T2 (1785), and CK (2363). As depicted in Figure 4 middle image, the number of overlapped OTUs among the groups R3, R9, R7, and CK decreased to seven. This indicates that the treatment with a mixture of cysteine and the microbial inoculant significantly altered the structure of the microbial communities in the paddy soils. The abundances of unique OTUs observed in both R3 (466) and R7 (499) were notably lower compared with those in R9 (2468) and CK (2809). This suggests that the additive amount of cysteine in the mixture had an impact on the microbial community structure and diversity. As depicted at Figure 4 lower image, the Venn diagram revealed six core OTUs between the CK and treatment groups (T1, T2, T3, R3, R7, R9). This indicates that distinct microbial communities were present in association with various leaching agents, as evidenced by a greater number of unique features compared with the core features.

**Figure 5:**
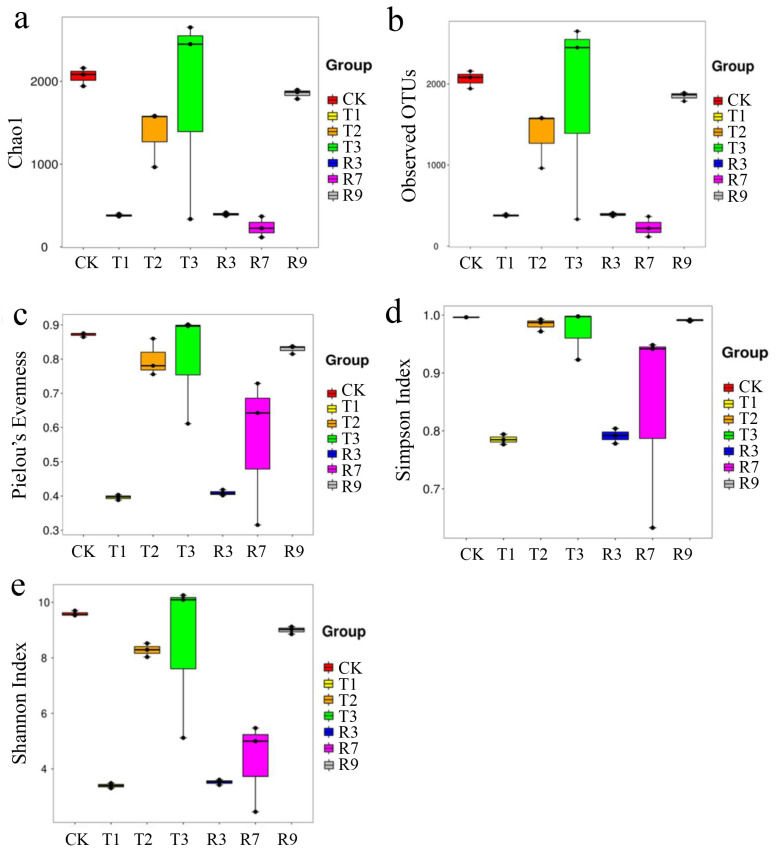
The representative alpha diversity indices, namely, Chao 1 (**a**), observed OTUs (**b**), Pielou’s evenness (**c**), and Simpson index (**d**), and Shannon index (**e**) after the different treatments (*p* < 0.05).

As shown in Figure 5, the α diversity of CK and the various treatment groups were assessed by the Simpson index, Shannon index, Pielou’s Evenness, and Chao 1 index. In all the samples, the observed coverage exceeded 99%, indicating that the abundance analysis provided an almost complete and reliable representation of the microbial diversity. The number of observed OTUs in CK (2059) was higher than those in the treatment paddy soils (234–1848.33). The values of the Simpson index, Shannon index, Pielou’s Evenness, and Chao 1 in CK were also higher than those in T1, T2, R5, R7, and R9. This suggested that due to the existence of cysteine and the microbial inoculant, the paddy soils became acidic, potentially leading to a decline in the soil microbial diversity. However, the diversity index in the sequential treatment groups (T1, T2, T3) were, on average, higher than in the combination groups (R3, R7, R9). This means that the different combinations of cysteine and microbial inoculant had impacts on the soil microbial diversity.

#### 3.6.2. Microbial Community Composition

**Figure 6:**
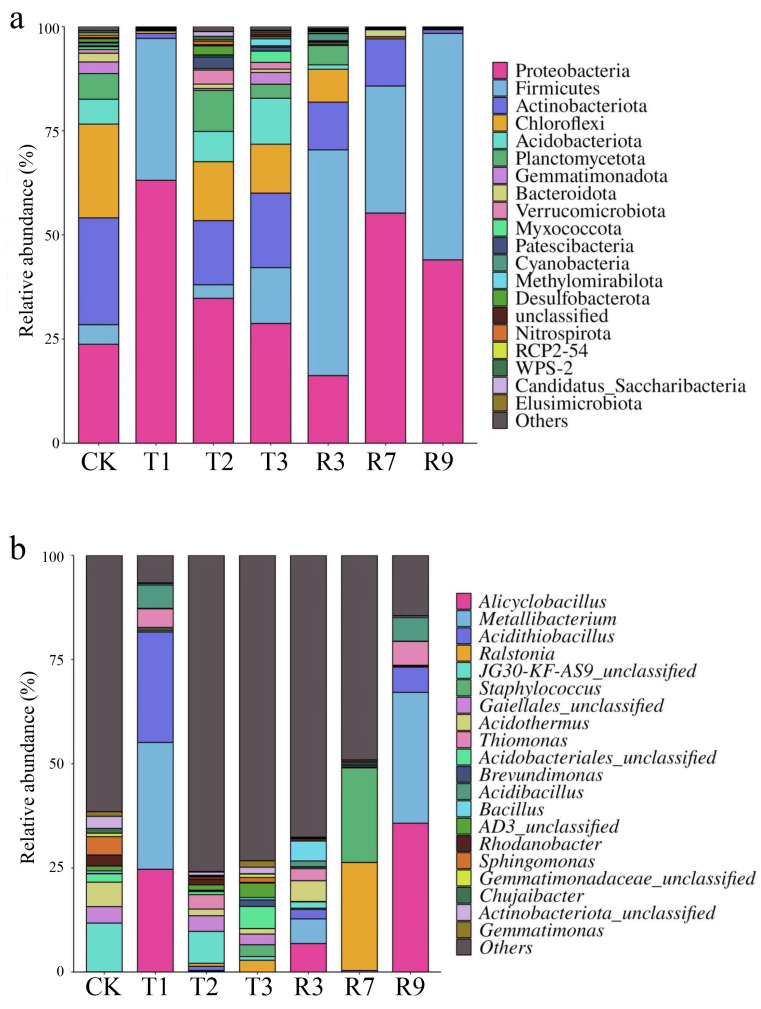
Relative abundances of bacterial community compositions after treatment at phylum (**a**) and genus (**b**) levels.

In order to delve deeper into the alterations in the bacterial community structure within paddy soils subsequent to treatment, we conducted a comprehensive analysis of the microbial composition at both the phylum and genus levels. As depicted in Figure 6a, Actinobacteriota (25.7%), Proteobacteria (23.8%), Chloroflexi (22.5%), Planctomycetota (6.2%), and Acidobacteriota (6.0%) were the dominant phyla in the original soil. Compared with the original soil, the relative abundances of Firmicutes and Proteobacteria were conspicuously elevated in the treatment groups. It has been reported that Proteobacteria and Firmicutes exhibit a high tolerance to heavy metals in paddy soils subjected to prolonged Cd pollution [22,65,66,67]. Moreover, their pivotal contribution to the biogeochemical cycles of carbon, nitrogen, and sulfur in soil rendered them environmentally indispensable [66]. The relative abundances of Planctomycetota and Verrucomicrobiota increased in T2 but decreased in the other treatment groups. The application of the microbial inoculant in T1 resulted in a significantly higher abundance of Proteobacteria (63.1%) that exceeded the proportions observed in T2 (34.8%) and T3 (28.7%). This might have been due to the microbial inoculant inherently containing microorganisms belonging to the phylum Proteobacteria, thereby resulting in a significant increase in Proteobacteria abundance in T1. Firmicutes were prevalent in the paddy soils after the R3, R7, and R9 treatments, which accounted for 54.2%, 30.5%, and 54.4% in these groups, respectively. This suggested that Firmicutes possessed remarkable adaptability to acidic conditions, and they were also found in the acidic mine drainage from a copper mine in India [68]. Additionally, the relative abundances of Bacteroidota, Chloroflexi, and Actinobacteria were decreased in each treatment group. This suggests that these microorganisms might be not suited to surviving in acidic or Cd-contaminated soil.

At the genus level, *JG30–KF–AS9_unclassified*, *Acidothermus*, *Sphingomonas*, *Gaiellales_unclassified*, *Actinobacteriota_unclassified*, and *Rhodanobacter* predominated in the original soil (Figure 6b). The microbial community of the paddy soils was significantly reshaped after the selected treatments. The dominant genera in T1 and R9 were notably similar, consisting primarily of *Metallibacterium*, *Alicyclobacillus*, *Acidithiobacillus*, *Staphylococcus*, and *Thiomonas*. This indicated that these microorganisms generally had good adaptability to the acidic environments and could thrive widely in acidic soils. Other studies also reported that *Metallibacterium*, *Alicyclobacillus*, *Acidithiobacillus*, and *Thiomonas* were widespread in acidic mine waters, acidic soils, and environments containing metal sulfides [69,70,71,72]. Moreover, the relative abundance of *Acidithiobacillus* in T1 was higher. This was due to the high concentration of microbial inoculants in T1, which introduced more of the *Acidithiobacillus* genus, and thus, resulted in a higher relative abundance of *Acidithiobacillus* in T1 after the treatment. *Acidithiobacillus* are able to use sulfides as electron donors and oxidize sulfur to sulfate. This process promotes the formation of acidic environments, thereby assisting in the dissolution of heavy metal ions from ores and soils [73]. And the dominant genera in T2 were *JG30–KF–AS9_unclassified* and *Gaiellales_unclassified*. The dominant genus of T3 was *Acidobacteriales_unclassifed*. The relative abundances of *Alicyclobacillus*, *Metallibacterium*, and *Bacillus* increased in R3 compared with CK. *Alicyclobacillus* can utilize sulfur as an energy source, which gives them a competitive advantage in sulfur-rich environments [74]. Additionally, it has been reported that *Alicyclobacillus* can participate in the degradation of organic matter and the transformation of minerals, contributing to improving the soil structure [75,76]. *Metallibacterium* exhibits high metal tolerance, enabling them to survive in heavy-metal-contaminated soils. They play important roles in the adsorption, transport, and redox reactions of heavy metal processes that contribute to decreasing the heavy metal toxicity in soil [77,78,79]. Additionally, *Metallibacterium* possesses diverse nitrogen, phosphorus, and sulfur metabolic pathways, which contributes to balancing these elements in soil, promoting plant growth, and enhancing the health of soil ecosystems [80,81,82]. *Bacillus* also exhibits a high tolerance to heavy metals [83]. *Bacillus* can regulate the concentration of heavy metal ions inside and outside cells through adsorption and transport mechanisms, thereby reducing their toxic effects on cells [84,85]. Additionally, *Bacillus* generally possesses strong capabilities to decompose and utilize organic substances. They can cause soils to release nutrients such as carbon, nitrogen, and phosphorus by decomposing organic matter, which are essential for the growth of soil microbes and plants [86,87,88]. In addition, R3, R7, and R9 exhibited significant differences in the relative abundances of dominant genera. This suggests that the concentrations of cysteine and the microbial inoculant could influence the microbial compositions after the treatments. The R3 group with a lower concentration of microbial inoculant exhibited a higher evenness between the dominant genera.

#### 3.6.3. Prediction of Microbial Metabolic Functions

To better understand the possible functions within the soil bacterial community, the bacterial metabolic functions in T1, T2, T3, R3, R7, and R9 were further analyzed. As seen in Figure S2, the relative abundances of sequences related to metabolism were the most dominant pathways in T1, T2, T3, R3, R7, and R9. This indicates that the introduction of elements such as C, S, and N by different leaching agents could promote the metabolism of the microbial community in paddy soils. In addition, organismal systems, cellular processes, environmental information processing, and genetic information processing were also found. The proportion of sequences related to organismal systems was the least, which was <1.5%. The relative abundance of cellular processes increased in almost all the treatment groups (T1, T2, R3, R7, R9) in comparison with CK. And the relative abundance of cellular processes in R3 could reach up to 6.3%. In addition, the high relative abundance of environmental information processing and genetic information processing were also observed for R3.

**Figure 7:**
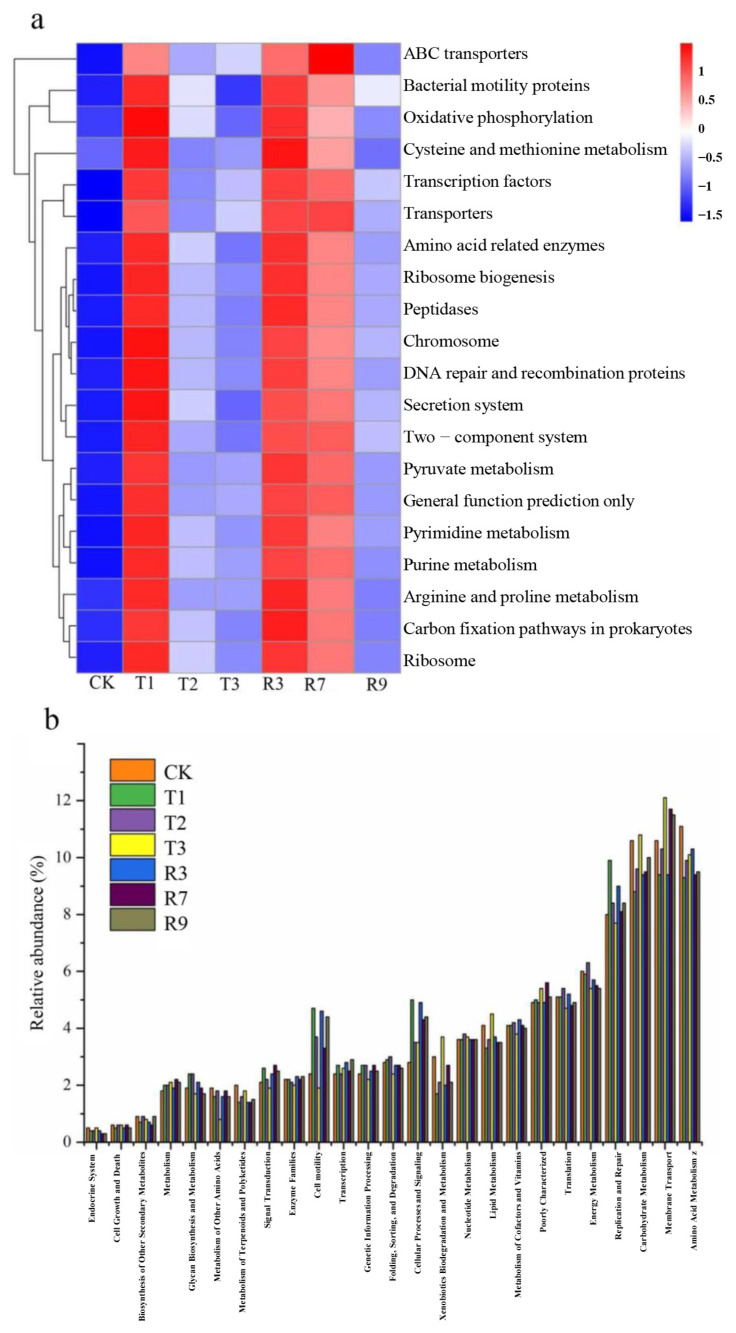
Bacterial metabolic functional pathways in selected soil samples at level 2 (**a**) and level 3 (**b**).

As shown in Figure 7a, the relative abundances of KOs at level 2 were analyzed to obtain a more profound comprehension of the functional differences. It was found that most of the relatively abundant functional genes belonged to metabolism at level 1. Compared with CK, the relative abundance of metabolism level 2, such as bacterial motility proteins, oxidative phosphorylation, cysteine and methionine metabolism, amino acid related enzymes, DNA repair and recombination proteins, and carbon fixation pathways in prokaryotes, were upregulated only in R3. This might be attributed to the increase in some nutrients and the change in pH value in the paddy soils after the R3 treatment. Figure 7b shows the prediction of microbial metabolic function at KEGG level 3. The relative abundances of amino acid metabolism were 9.3%, 9.9%, 10.1%, 10.3%, 9.45%, and 9.5% for T1, T2, T3, R3, R7, and R9, respectively. And this was the predominant pathway in the cluster of metabolisms. Furthermore, the relative abundances of carbohydrate metabolism were 8.8%, 9.6%, 10%, 9.4%, 9.5%, and 10% for T1, T2, T3, R3, R7, and R9, respectively. And the energy metabolisms could reach up to 5.9%, 6.3%, 5.4%, 5.7%, 5.5%, and 5.4% for T1, T2, T3, R3, R7, and R9, respectively. In the sequence related to the genetic information processing, the relative abundance of replication and repair was higher in R3 when compared with CK. And the amino acid metabolism was the dominant pathways in R3. This indicates that the R3 treatment could promote the bacterial metabolisms, such as replication and repair and amino acid metabolism, to maintain the paddy soil health.

## 4. Conclusions

In this study, the combination of cysteine and a microbial inoculant on the bioremediation of Cd contaminated paddy soils was investigated. The results show that the mixture of cysteine and the microbial inoculant was more effective at removing Cd from the contaminated paddy soils. The rate of increase in the volume of the additive amount of cysteine increased with the removal rate. The mixture of 200 mL of microbial inoculant and 0.25 g of cysteine was found to have the highest economic efficiency. And the Cd removal rate was in the range of 54.05–85.95%, which was 7.65–15.05% higher than the removal rate in the microbial inoculant treatment. The mixture of cysteine and the microbial inoculant was more effective at removing the F1 and F2 fractions of Cd, where they achieved removal rates of 94.56% and 96.11%, respectively. The contents of NH_3_–N, TP, AK, and AP showed notable increases in the paddy soils following the treatment. Due to the addition of cysteine and the microbial inoculant, the microbial diversities in the paddy soils were affected. The relative abundances of genera such as *Alicyclobacillus*, *Metallibacterium*, and *Bacillus* increased, which potentially promoted the carbon, nitrogen, phosphorus, and sulfur metabolisms in the paddy soils. The relative abundances of microbial metabolic functions, including replication and repair and amino acid metabolism, were significantly promoted, which was conducive to microbial survival and soil health. The removal of Cd was attributed to the solubilization, complexation, and ion-exchange effects of cysteine, as well as cysteine as a substrate to provide energy for the microorganisms to promote the growth and metabolism, which enhanced the microorganisms’ removal of Cd through the metabolism of organic acids, intracellular adsorption, and other effects.

## Figures and Tables

**Table 1:** Experimental groupings of cysteine and microbial inoculants for leaching Cd from paddy soil.

Group	Order of Addition
T1	Microbial inoculant–microbial inoculant–microbial inoculant
T2	Cysteine solution–cysteine solution–cysteine solution
T3	Microbial inoculant–cysteine solution–microbial inoculant
T4	Microbial inoculant–cysteine solution–cysteine solution
T5	Cysteine solution–microbial inoculant–microbial inoculant
T6	Microbial inoculant–microbial inoculant–cysteine solution
T7	Cysteine solution–microbial inoculant–cysteine solution
T8	Cysteine solution–cysteine solution–microbial inoculant
T9	A mixed solution of cysteine and microbial inoculant–mixed solution of cysteine and microbial inoculant–mixed solution of cysteine and microbial inoculant

**Table 2:** Experimental groupings of microbial inoculant and cysteine ratio for Cd leaching from paddy soil.

Groups	Ratio of Cysteine to Microbial Inoculant
R1	100 mL of microbial inoculant with a dose of 0.1 g of cysteine was diluted 3-fold with water to 300 mL
R2	100 mL of microbial inoculant with a dose of 0.15 g of cysteine was diluted 3-fold with water to 300 mL
R3	100 mL of microbial inoculant with a dose of 0.25 g of cysteine was diluted 3-fold with water to 300 mL
R4	150 mL of microbial inoculant with a dose of 0.1 g of cysteine was diluted 3-fold with water to 300 mL
R5	150 mL of microbial inoculant with a dose of 0.15 g of cysteine was diluted 3-fold with water to 300 mL
R6	150 mL of microbial inoculant with a dose of 0.25 g of cysteine was diluted 3-fold with water to 300 mL
R7	200 mL of microbial inoculant with a dose of 0.1 g of cysteine was diluted 3-fold with water to 300 mL
R8	200 mL of microbial inoculant with a dose of 0.15 g of cysteine was diluted 3-fold with water to 300 mL
R9	200 mL of microbial inoculant with a dose of 0.25 g of cysteine was diluted 3-fold with water to 300 mL

**Table 3:** Selected paddy soil physicochemical properties before and after treatment (T1–T3).

Parameter	S1				S2				S3			
	Ini-Soil	T1	T2	T3	Ini-Soil	T1	T2	T3	Ini-SOIL	T1	T2	T3
pH	4.54 ± 0.15 a	3.11 ± 0.01 b	2.80 ± 0.01 c	2.91 ± 0.01 c	4.51 ± 0.02 a	1.96 ± 0.06 d	3.04 ± 0.05 c	2.01 ± 0.01 d	5.06 ± 0.24 a	3.59 ± 0.01 c	3.70 ± 0.03 c	2.86 ± 0.02 d
SOM (%)	1.86 ± 0.07 a	0.66 ± 0.05 c	0.53 ± 0.03 c	1.10 ± 0.03 b	2.75 ± 0.25 a	2.37 ± 0.07 b	0.94 ± 0.06 e	2.62 ± 0.15 b	3.99 ± 0.49 a	1.09 ± 0.03 c	0.50 ± 0.07 e	1.84 ± 0.28 b
TN (g/kg)	2.52 ± 0.11 ab	1.32 ± 0.03 c	1.17 ± 0.04 c	2.22 ± 0.35 b	2.65 ± 0.25 a	2.53 ± 0.11 ab	1.57 ± 0.03 d	2.02 ± 0.10 c	2.99 ± 0.14 a	2.08 ± 0.04 a	1.80 ± 0.12 c	2.02 ± 0.05 b
NH_3_–N (mg/kg)	16.36 ±3.67 d	872.0 ± 20.8 b	32.53 ± 1.73 c	1269.09 ± 193 a	25.39 ± 0.96 f	1071.3 ± 53.1 a	259.5 ± 26.7 e	780.36 ± 9.45 c	13.67 ± 1.32 c	474.2 + 45.1 b	419.6 ± 48.3 b	711.76 ± 32.80 a
NO_3_–N (mg/kg)	31.13 ± 0.40 e	44.30 ± 0.91 d	63.09 ± 3.33 b	58.72 ± 1.09 b	51.00 ± 1.21 c	48.82 ± 2.65 c	36.94 ± 3.86 d	67.91 ± 1.53 b	57.89 ±0.98 b	64.23 ± 3.53 ab	59.06 ± 2.42 b	69.95 ± 2.26 a
TP (g/kg)	0.43 ± 0.01 c	0.34 ± 0.02 c	0.29 ± 0.01 c	0.55 ± 0.07 b	0.33 ± 0.03 f	0.75 ± 0.03 a	0.37 ± 0.03 e	0.46 ± 0.02 c	0.47 ± 0.01 b	0.55 ± 0.04 a	0.35 ± 0.02 c	0.59 ± 0.01 a
AK (mg/kg)	136.04 ± 4.23 f	366.88 ± 2.23 c	49.2 ± 0.34 f	561.51 ± 23.57 a	130.61 ± 1.70 e	501.9 ± 23.2 a	104.38 ± 2.04 f	125.93 ± 3.45 f	102.51 ± 1.00 e	236.68 ± 6.02 b	62.09 ± 5.69 e	345.83 ± 3.50 a
AP (mg/kg)	25.29 ± 1.29 c	61.64 ± 3.92 b	31.30 ± 1.96 c	7.84 ± 1.29 d	8.47 ± 1.18 bc	18.94 ± 1.01 a	15.26 ± 2.77 a	1.85 ± 0.28 d	11.38 ± 1.02 d	7.75 ± 0.72 d	26.02 ± 3.60 c	5.35 ± 0.12 d

Different letters in the same line suggest a significant difference (*p* < 0.05) between soil samples.

**Table 4:** Selected paddy soil physicochemical properties before and after treatment (R3, R7, R9).

Parameter	S1				S2				S3			
	Ini-Soil	R3	R7	R9	Ini-Soil	R3	R7	R9	Ini-Soil	R3	R7	R9
pH	4.54 ± 0.15 a	3.28 ± 0.05 b	2.74 ± 0.04 c	2.83 ± 0.05 c	4.51 ± 0.02 a	3.49 ± 0.02 b	2.08 ± 0.01 d	3.40 ± 0.16 b	5.06 ± 0.24 a	4.00 ± 0.01 b	2.67 ± 0.01 d	2.10 ± 0.01 e
SOM (%)	1.86 ± 0.07 a	0.72 ± 0.00 c	0.76 ± 0.02 c	0.96 ± 0.02 c	2.75 ± 0.25 a	1.60 ± 0.03 c	2.35 ± 0.08 b	1.34 ± 0.05 d	3.99 ± 0.49 a	0.66 ± 0.04 de	0.84 ± 0.04 cd	0.90 ± 0.01 cd
TN (g/kg)	2.52 ± 0.11 ab	2.10 ± 0.05 b	2.23 ± 0.09 b	2.80 ± 0.11 a	2.65 ± 0.25 a	2.09 ± 0.02 bc	1.57 ± 0.01 d	2.27 ± 0.09 b	2.99 ± 0.14 a	2.04 ± 0.11 b	1.85 ± 0.06 bc	1.68 ± 0.02 c
NH_3_–N (mg/kg)	16.36 ±3.67 d	675.49 ± 16.89 b	738.7 ± 20.4 b	1121.7 ± 74.9 a	25.39 ± 0.96 f	558.8 ± 34.3 d	542.23 ± 6.37 d	898.3 ± 33.9 b	13.67 ± 1.32 c	510.7 ± 39.5 b	515.6 ± 30.3 b	449.0 ± 36.4 b
NO_3_–N (mg/kg)	31.13 ± 0.40 e	46.90 ± 0.33 cd	49.43 ± 1.77 cd	52.35 ± 3.23 c	51.00 ± 1.21 c	51.98 ± 1.06 c	103.14 ± 5.39 a	43.02 ± 1.03 cd	57.89 ±0.98 b	62.79 ± 2.06 ab	49.43 ± 1.77 c	52.02 ± 0.31 c
TP (g/kg)	0.43 ± 0.01 c	0.59 ± 0.04 b	0.60 ± 0.03 b	0.75 ± 0.03 a	0.33 ± 0.03 f	0.57 ± 0.01 c	0.51 ± 0.00 d	0.64 ± 0.01 b	0.47 ± 0.01 b	0.48 ± 0.03 b	0.45 ± 0.02 b	0.45 ± 0.02 b
AK (mg/kg)	136.04 ± 4.23 f	261.30 ± 2.03 e	319.64 ± 15.91 d	422.8 ± 22.0 b	130.61 ± 1.70 e	284.78 ± 1.10 c	214.50 ± 2.20 d	412.88 ± 4.84 b	102.51 ± 1.00 e	226.29 ± 4.94 bc	205.1 ± 17.8 c	109.63 ± 6.34 d
AP (mg/kg)	25.29 ± 1.29 c	72.10 ± 3.37 a	71.24 ± 5.90 a	73.03 ± 2.39 a	8.47 ± 1.18 bc	8.49 ± 3.90 bc	14.54 ± 2.34 ab	4.48 ± 2.42 c	11.38 ± 1.02 d	34.88 ± 1.58 b	56.50 ± 2.92 a	28.23 ± 0.16 c

Different letters in the same line suggest a significant difference (*p* < 0.05) between soil samples.

## Data Availability

The original data presented in this study are included in this article; further inquiries can be directed to the corresponding author.

## Supplementary Materials

The following supporting information can be downloaded at: https://www.mdpi.com/article/10.3390/toxics13010022/s1, Figure S1: Economic benefits analysis of different treatments; Figure S2: Microbial metabolic functional pathways in selected soil samples at level 1; Table S1: Steps in the determination of Cd fractionation in the soil by Tessier sequential extraction method.
