# Supplementary material for: The Effect of Cysteine on the Removal of Cadmium in Paddy Soil by Combination with Bioremediation and the Response of the Soil Microbial Community
Source: Toxics. 2024 Dec 29;13(1):22. doi: 10.3390/toxics13010022 (PMC11769394; doi:10.3390/toxics13010022)
Supplement: Supplementary file 1 [file toxics-13-00022-s001.zip › toxics-3351720-supplementary.pdf]

toxics

MDPI

Article

The Effect of Cysteine on the Removal of Cadmium in Paddy Soil by Combination with Bioremediation and the Response of the Soil Microbial Community

Emmanuel Konadu Sarkodie [1,2], Kewei Li [1,2], Ziwen Guo [1,2], Jiejie Yang [1,2], Yan Deng [3], Jiaxin Shi [1,2], Yulong Peng [1,2], Yuli Jiang [1,2], Huidan Jiang [3], Hongwei Liu [1,2], Yili Liang [1,2], Huaqun Yin [1,2], Xueduan Liu [1,2] and Luhua Jiang [1,2,*]

[1] School of Minerals Processing and Bioengineering, Central South University, Changsha 410083, China; emmasarkk@gmail.com (E.K.S.); 15116475510@163.com (K.L.); kidgzw@hotmail.com (Z.G.); jiejieyang@csu.edu.cn (J.Y.); 18874761977@163.com (J.S.); 225611009@csu.edu.cn (Y.P.); 235611001@csu.edu.cn (Y.J.); hongweiliu@csu.edu.cn (H.L.); liangyili6@csu.edu.cn (Y.L.); yinhuaqun_cs@sina.com (H.Y.); xueduanliu@csu.edu.cn (X.L.)

[2] Key Laboratory of Biometallurgy of Ministry of Education, Central South University, Changsha 410083, China

[3] Hunan Agricultural Biotechnology Research Institute, Hunan Academy of Agricultural Sciences, Changsha 410125, China; dengyan202103@163.com (Y.D.); jianghuidan@hunaas.cn (H.J.)

* Correspondence: jiangluhua@csu.edu.cn

Table S1 Steps in the determination of Cd fractionation in the soil by Tessier sequential extraction method

Step	Fraction	Operation sequence
1	Exchangeable fraction (F1)	8 mL of 1 mol/L magnesium chloride solution was added into 1.0 g soil in a 50 mL centrifuge tube, shaking at room temperature for 1 h (200 r/min) and then centrifuging for 10 min (4000 r/min)
2	Carbonate bound fraction (F2)	The residue from step 1 was leached with 8 mL of 1 mol/L sodium acetate at room temperature. Before extraction, the pH was adjusted to 5.0 with acetic acid, shaking for 8 h (200 r/min) and then centrifuging for 10 min (4000 r/min)
3	Iron/Manganese bound fraction (F3)	20 mL of 0.04 mol/L hydroxylamine hydrochloride in 25% (v/v) acetic acid solution was added into the residue after step 2 for extraction. The extraction temperature was 96 ± 3 °C with occasional agitation for 4 h. Centrifugation was done for 10 min (4000 r/min) and the supernatant removed and filtered
4	Organic matter bound fraction (F4)	3 mL of 0.02 mol /L nitric acid and 5 mL of 30% (V/V) hydrogen peroxide was added into the residue treated at step 3. The pH was adjusted to 2 with nitric acid, and

		then the mixture was heated at 85 ± 2 °C for 2 h with occasional agitation. After that, 5 mL hydrogen peroxide was added and the pH was adjusted to 2. Then, samples were heated again at 85 ± 2 °C for 3 h and shaken intermittently. After cooling, 5 mL of 3.2 mol/L ammonium acetate in 20% (V/V) nitric acid was added and diluted to 20 mL and then shaken for 30 min. Centrifuge was done for 10 min (4000 r/min), supernatant removed, and the removed solution filtered. 50 mL volumetric flask was used to make the volume constant
5	Residual fraction (F5)	The residue from step 4 was digested and analyzed by the nitric acid-hydrofluoric acid-perchloric acid digestion method

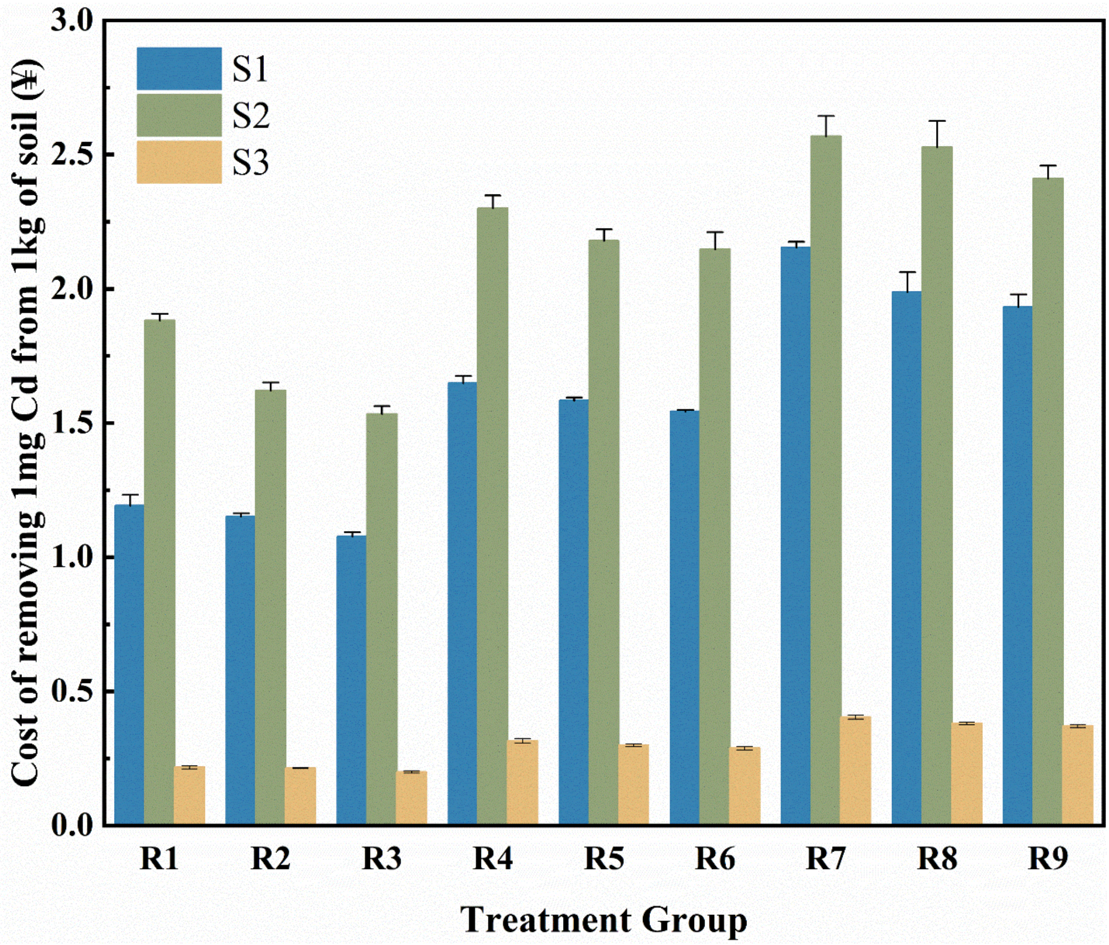

Figure S1 Economic benefits analysis of different treatments

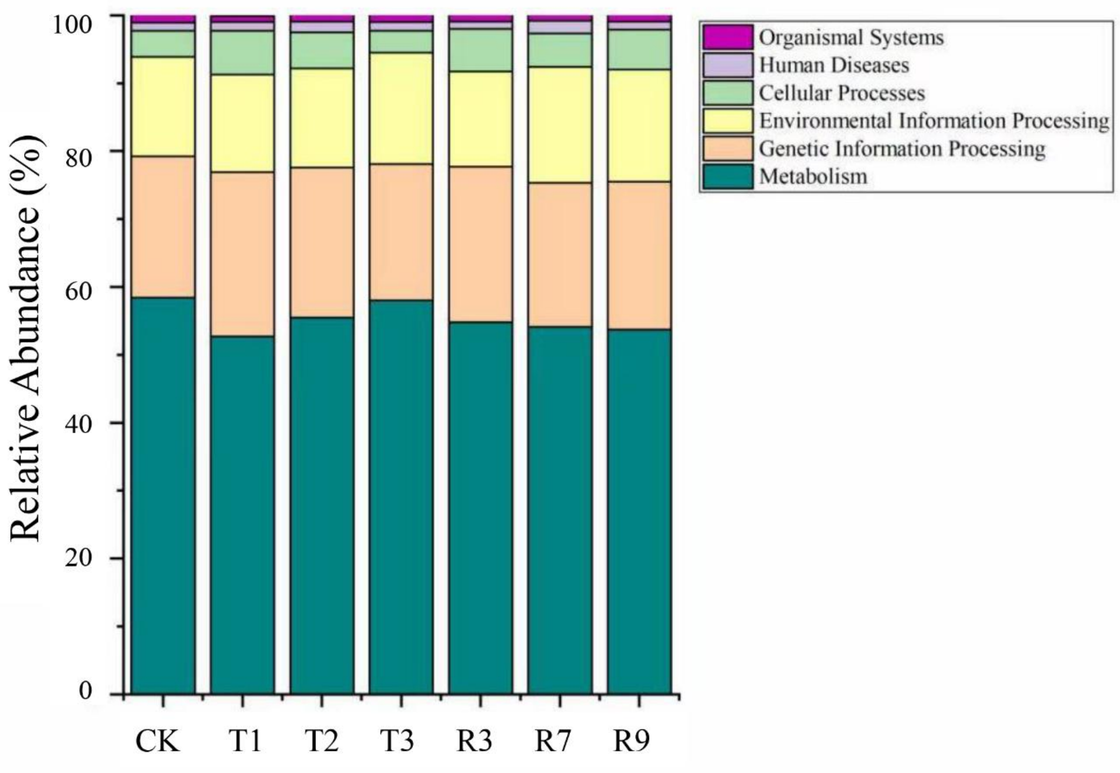

Figure S2 Microbial metabolic functional pathways in selected soil samples at level 1
